# Role of gasdermin D in inflammatory diseases: from mechanism to therapeutics

**DOI:** 10.3389/fimmu.2024.1456244

**Published:** 2024-08-26

**Authors:** Chak Kwong Cheng, Min Yi, Li Wang, Yu Huang

**Affiliations:** ^1^ Department of Biomedical Sciences, City University of Hong Kong, Hong Kong, Hong Kong SAR, China; ^2^ Department of Endocrinology, The Second Affiliated Hospital of Guangzhou Medical University, Guangzhou, China

**Keywords:** cell death, gasdermin D, GSDMD inhibitor, inflammation, inflammatory diseases, necroptosis, NEtosis, pyroptosis

## Abstract

Inflammatory diseases compromise a clinically common and diverse group of conditions, causing detrimental effects on body functions. Gasdermins (GSDM) are pore-forming proteins, playing pivotal roles in modulating inflammation. Belonging to the GSDM family, gasdermin D (GSDMD) actively mediates the pathogenesis of inflammatory diseases by mechanistically regulating different forms of cell death, particularly pyroptosis, and cytokine release, in an inflammasome-dependent manner. Aberrant activation of GSDMD in different types of cells, such as immune cells, cardiovascular cells, pancreatic cells and hepatocytes, critically contributes to the persistent inflammation in different tissues and organs. The contributory role of GSDMD has been implicated in diabetes mellitus, liver diseases, cardiovascular diseases, neurodegenerative diseases, and inflammatory bowel disease (IBD). Clinically, alterations in GSDMD levels are potentially indicative to the occurrence and severity of diseases. GSDMD inhibition might represent an attractive therapeutic direction to counteract the progression of inflammatory diseases, whereas a number of GSDMD inhibitors have been shown to restrain GSDMD-mediated pyroptosis through different mechanisms. This review discusses the current understanding and future perspectives on the role of GSDMD in the development of inflammatory diseases, as well as the clinical insights of GSDMD alterations, and therapeutic potential of GSDMD inhibitors against inflammatory diseases. Further investigation on the comprehensive role of GSDM shall deepen our understanding towards inflammation, opening up more diagnostic and therapeutic opportunities against inflammatory diseases.

## 1 Introduction

As an evolutionarily conserved biological response, inflammation primarily confers host protection against pathogen insults and promotes tissue repair by activating both immune and non-immune cells. An unfavorable, prolonged, and persistent inflammation, either locally or systemically, characterizes inflammatory diseases (1). Inflammatory diseases are recognized as one of the leading causes of mortality worldwide today, where nearly 50% of all-cause mortality is attributed to inflammation-associated complications, like carcinomas, ischemic heart disease, diabetes mellitus, autoimmune diseases, and neurodegenerative disorders (2).

**Figure 1:**
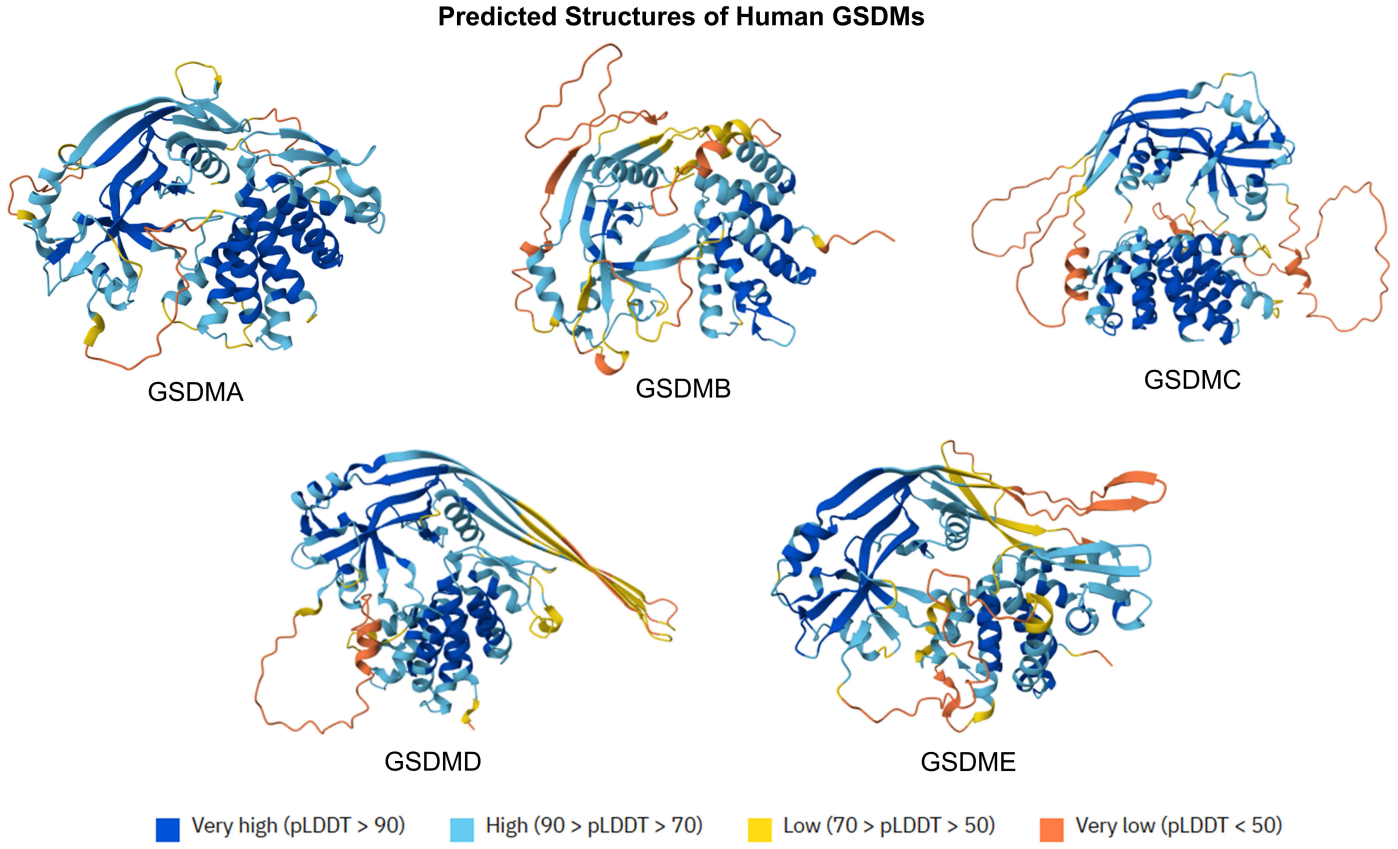
Predicted protein structures of human GSDMs. The predicted structures of GSMD family (GSDMA to GSDME) are displayed at orient axes. The predicted protein structures are generated by AlphaFold 3. AlphaFold produces a per-residue model confidence score (pLDDT) between 0 and 100.

The gasdermin (GSDM) family is constituted by five members (GSDMA to GSDME), representing a group of lipid-binding and membrane pore-forming proteins. Primarily characterized as mediators of pyroptotic cell death, GSDMs are shown to also mediate vital cell functions, non-lytic release of inflammatory cytokines, and bactericidal action (3). GSDMs are constituted by N-terminal domains (NTDs), which can initiate cytolysis by forming membrane pores, and C-terminal domains (CTDs), which can inhibit cell death via intramolecular domain association (4). The predicted structures of human GSDMs are displayed in **Figure 1**. Belonging to the GSDM family, gasdermin D (GSDMD) is the most extensively studied GSDM, which is a critical modulator of inflammasome-dependent pyroptosis and diverse downstream immunological activities (5). Both human and murine GSDMDs are composed of NTDs and CTDs, interconnected by a partially disordered linker region. Suggested by crystal structure study, no gross structural alterations were noted across both NTDs and CTDs from human and murine GSDMDs (6).

Briefly, activated by various inflammasomes, GSDMD is tightly involved in the execution of pyroptosis, downstream inflammatory signaling cascades, and the release of inflammatory cytokines. Aberrant GSDMD activation triggers persistent inflammation and has been linked to the development of numerous inflammatory diseases, such as diabetes mellitus, liver diseases, cardiovascular diseases, neurodegenerative diseases, inflammatory bowel disease (IBD), and sepsis (7). This review article discusses the relationship between GSDMD and cell death, the mechanisms of GSDMD-related inflammatory signal transduction, and the role of GSDMD in different cell types. In addition, the role of GSDMD in different inflammatory diseases, clinical insights of alterations in GSDMD levels, and the therapeutic potential of GSDMD inhibitors are also addressed.

## 2 Role of GSDMD in distinct types of cell death

**Figure 2:**
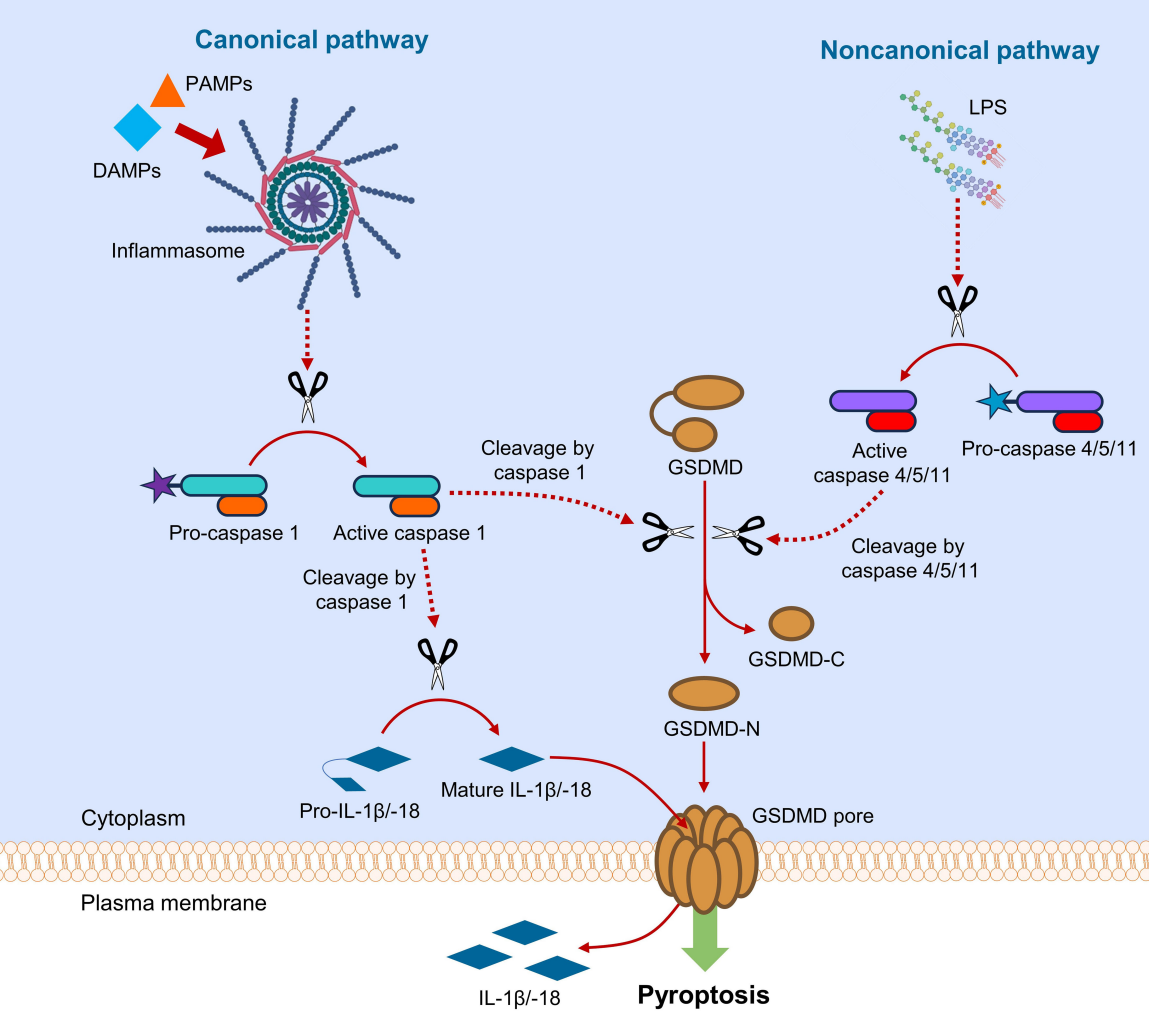
Canonical and noncanonical pathways of GSDMD-mediated pyroptosis. In canonical pathway, sensing DAMPs and PAMPs by inflammasome triggers downstream activation of caspase 1, which cleaves GSDMD and pro-IL-1β/-18. In noncanonical pathway, LPS from Gram-negative bacteria activates caspase 4/5/11, which subsequently cleaves GSDMD. Upon the action of caspases, GSDMD is cleaved into fragments containing NTD and CTD (GSDMD-N and GSDMD-C). GSDMD-N assembles to cause pore formation on plasma membrane, leading to the release of interleukins.

Although GSDMD is primarily considered as the critical gatekeeper for igniting pyroptotic cell death (**Figure 2**), GSDMD also participates in the mediation of other types of cell death, namely apoptosis, necroptosis and NETosis.

### 2.1 GSDMD-mediated pyroptosis

Pyroptosis is considered an inflammatory form of programmed cell death, ignited by host factors or pathogenic infections (8). Pyroptosis can be initiated by canonical or noncanonical pathway, where GSDMD serves as a common downstream effector for both pathways (**Figure 2**) (9). In canonical pyroptotic pathway, pattern recognition receptors (PRRs) are intracellularly activated by damage-associated molecular patterns (DAMPs) and pathogen-associated molecular patterns (PAMPs) in cytoplasm, where activated PRRs assemble apoptosis-associated speck-like protein (ASC) and pro-caspase 1 to form a multi-protein complex termed inflammasome. The activated caspase 1 is responsible for the cleavage of GSDMD and pro-IL-1β/IL-18 to their mature forms (10). In noncanonical pathway, intracellular lipopolysaccharides (LPS) from Gram-negative bacteria activate pro-caspase 4/5 (in human) or pro-caspase 11 (in murine) (11). The active caspase 4/5/11 subsequently cleaves the linker region in GSDMD to produce NTD- and CTD-containing fragments (GSDMD-N and GSDMD-C) for pyroptosis initiation (12). The NTD-containing GSDMD fragment (GSDMD-N) oligomerizes to form transmembrane pore by selectively interacting with membrane lipids, resulting in the release of danger signals and cellular contents, especially inflammatory cytokines (13). Crosstalk between canonical and noncanonical pyroptotic pathways exists through NOD-like receptor family pyrin domain containing 3 (NLRP3) (14), which aggravates inflammation by activating caspase 1 for cleavage of pro-IL-1β/IL-18 into their mature forms (14, 15).

Additional to caspases, GSDMD can be cleaved by other proteases, such as neutrophil serine proteases (NSPs) (e.g. elastase and cathepsin G), and main protease (Mpro) from SARS-CoV-2 virus. In activated neutrophils, GSDMD can be cleaved by neutrophil elastase, where cleaved GSDMD plays a regulatory role in the generation of neutrophil extracellular traps (NETs), neutrophil pyroptosis, and inflammasome activation in neutrophils (16). Meanwhile, GSDMD can be cleaved by cathepsin G, another NSP. Cathepsin G cleaves GSDMD at leucine 274 to form the specific nitrogen-terminal domain p30, which is believed to play a critical role in regulating inflammatory responses and cell death pathways in neutrophils and monocytes (17). Mpro and papain-like protease (PLpro) are highly conserved viral proteases critical to the replication of SARS-COV-2. Recently, it was shown that Mpro can cleave porcine GSDMD at the glutamine 193-glycine 194 junction to generate two fragments, which are unable to cause pyroptosis. In other words, Mpro can cleave pore-forming p30 fragment of porcine GSDMD to antagonize GSDMD-mediated pyroptosis, and to facilitate the replication of coronaviruses (18). Further studies shall investigate the effects of other SARS-COV-2-derived proteases, like PLpro, on the structure and function of GSDMD.

### 2.2 GSDMD-mediated apoptosis

GSDMs (GSDMB to GSDME) are generally considered as negative regulators of apoptosis that the presence of their active forms generally switches apoptosis to pyroptosis to elicit inflammation. In other words, low levels of these GSDMs generally facilitate apoptosis over pyroptosis (19). GSDMD has been implicated to switch apoptosis to pyroptosis in numerous disease models. Caspase-1 is responsible for the cleavage of GSDMD to GSDMD-N and GSDMD-C fragments to induce pyroptosis. Nevertheless, caspase-1 was shown to be pro-pyroptotic in GSDMD-sufficient cells, but pro-apoptotic in GSDMD-deficient macrophages through the Bid-caspase-9-caspase-3 axis (20). In GSDMD-deficient macrophages, inflammasome activation was also shown to activate apoptotic caspase-3 and caspase-7 in a caspase-1/8-dependent manner (21).

**Figure 3:**
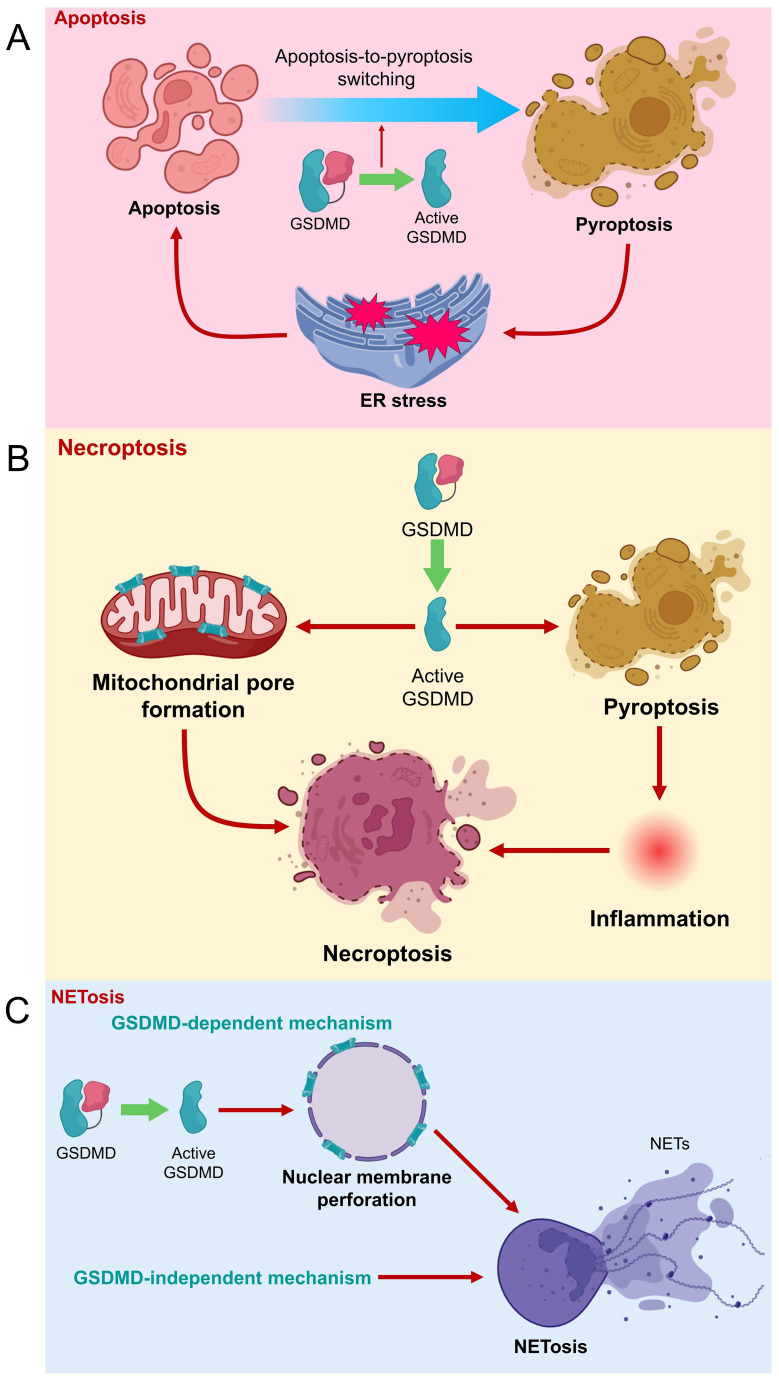
Role of GSDMD in different types of cell death. **(A)** GSDMD is suggested to be a negative regulator of apoptosis. However, GSDMD-induced pyroptosis can cause ER stress, which eventually triggers apoptosis. **(B)** GSDMD cleavage leads to pore formation on mitochondrial membrane to promote necroptosis. Meanwhile, GSDMD-mediated pyroptosis and inflammation are potentially upstream to necroptosis. **(C)** NETosis can be caused by GSDMD-dependent and -independent mechanisms. Active GSDMD (GSDMD-N) forms pores on nuclear membrane of neutrophil to the release of NETs and NETosis.

Moreover, GSDMD knockdown was found to promote apoptotic cell death and inhibit EGFR/Akt signaling in non−small cell lung cancer cells (22). Conversely, downstream to toll-like receptor 4 (TLR4), GSDMD upregulation was shown to elevate pyroptotic rate but reduce apoptotic rate in a proximal tubular cell to potentially aggravate tubular injury (23). Additionally, GSDMD deficiency was found to upregulate apoptosis during noninfectious liver injury (24). However, some recent findings have suggested controversial roles of GSDMD in mediating apoptosis (**Figure 3**). For instance, GSDMD overexpression was shown to enhance eIF2α phosphorylation and activate endoplasmic reticulum (ER) stress response to promote tumor cell apoptosis, rather than pyroptosis, during cisplatin chemotherapy (25). Another study even showed that GSDMD-induced pyroptosis is upstream to apoptosis in human neuroblastoma cells upon bisphenol A treatment, in a caspase-1-dependent manner (26). Further mechanistic studies are needed to investigate the crosstalk between pyroptosis and apoptosis, and the comprehensive role of GSDMD in relating inflammation, pyroptosis and apoptosis.

### 2.3 GSDMD-mediated necroptosis

Necroptosis is another mode of programmed cell death that shares certain apoptotic and necrotic features, where receptor-interacting protein kinase 3 (RIPK3) and its substrate mixed lineage kinase domain like pseudokinase (MLKL) are crucial executors of this pathway (27, 28). Recent findings have suggested a linkage between GSDMD-mediated pyroptosis and necroptosis (**Figure 3**). Weindel et al. showed that upon perturbed mitochondrial homeostasis, increased mitochondrial ROS guides GSDMD to form pores on mitochondrial membranes, releasing mitochondrial ROS to the cytoplasm. The intracellular mitochondrial ROS then facilitates the switch from pyroptosis to RIPK3/MLKL-dependent necroptosis (29). These findings imply that GSDMD might be closely involved in mitochondrial homeostasis, and strategies that guide GSDMD away from plasma membrane might retard pyroptosis. Furthermore, another study indicates that GSDMD-mediated pyroptosis and inflammation potentially precede necroptosis during intestinal inflammation, contributory to the development of colitis (30). Therefore, GSDMD might potentially be an executor of different cell death pathways, which requires further extensive studies to confirm.

### 2.4 GSDMD-mediated NETosis

NETosis refers to a special type of programmed cell death, involving the formation and release of NETs by dying neutrophils to counteract pathogenic insults (31). GSDMD has been demonstrated to mediate the generation of NETs by neutrophils, in which neutrophil proteases cause proteolytical activation of GSDMD, where activated GSDMD in turn modulates protease activation and nuclear expansion in a feed-forward loop (32). GSDMD cleavage was previously shown to be upstream to NET release in neutrophils during acute respiratory distress syndrome, where extrinsic NETs could significantly reverse the protection of GSDMD inhibition against mouse lung injury (33). GSDMD-mediated NET formation was also found positively associated with infiltration of inflammatory cells, macrophage-to-myofibroblast transition, and renal fibrosis during obstructive nephropathy (34). Mechanistically, GSDMD-N can form pores on nuclear and plasma membranes of neutrophils (35), causing the release of NETs from neutrophils (36). However, some recent studies proposed that GSDMD is unessential for NETosis initiation. Chauhan et al. provided clues that phorbol ester-induced NETosis still occurs even in GSDMD-deficient neutrophils (37). In 2023, Stojkov et al. indicated that NETosis could be triggered by inflammasome-independent mechanisms as GSDMD cleavage is not necessarily a preceding event for NET formation. In other words, NETs can be formed by viable neutrophils upon inflammasome activation independent of GSDMD (38). Therefore, the previously suggested concept that pyroptosis is a prerequisite for NET generation need revisit. However, it is also reasonable to postulate that NETosis might occur in both GSDMD-dependent and GSDMD-independent mechanisms, urging further detailed investigation.

## 3 Role of GSDMD in different cell types

**Figure 4:**
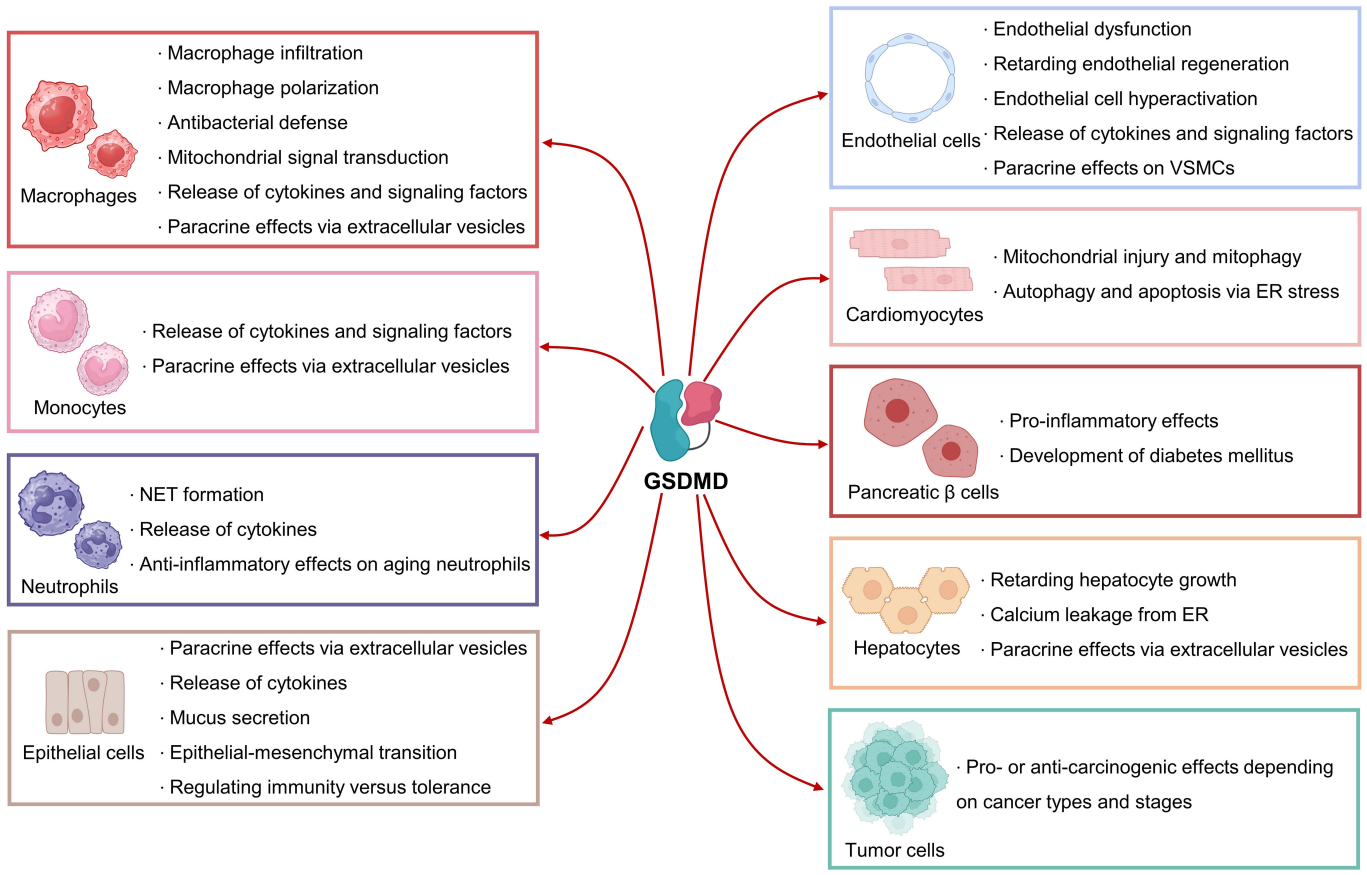
Biological roles of GSDMD in different cell types. GSDMD plays mediatory roles in the functions of immune cells, cardiovascular cells, pancreatic β cells, hepatocytes and tumor cells. GSDMD can exert pleiotropic effects depending on cell types and stages.

GSDMD-mediated pore formation, pyroptosis and inflammation have been implicated in different types of cells, such as immune cells, cardiovascular cells, epithelial cells, pancreatic β cells, and hepatocytes (**Figure 4**). Notably, GSDMD might elicit conflicting effects in different cell types, meaning that cell-specific therapies targeting GSDMD shall be taken into consideration in the context of different diseases.

### 3.1 Immune cells

GSDMD is actively involved in the mediation of pyroptosis in immune cells, particularly monocytes, macrophages, and neutrophils. GSDMD has been experimentally shown to modulate macrophage infiltration. Deletion of GSDMD remarkably retarded macrophage infiltration to the lesion sites in aortas during atherogenesis (39), hinting that GSDMD-mediated pyroptosis in aortic cells (e.g. resident immune cells and endothelial cells) might account for the release of chemokines. Besides, GSDMD is involved in the mediation of macrophage polarization towards M1 subtype during acute kidney injury (40). Whether GSDMD mediates macrophage polarization in a similar fashion in other tissues requires further investigation. Moreover, GSDMD is sensitive to mitochondrial ROS during mitochondrial dysfunction, where subsequent GSDMD oxidation represents a *de novo* mechanism driving macrophage pyroptosis (41). GSDMD-mediated pyroptosis and NETosis were found to coordinate antibacterial defense in macrophages (42). In addition to the pro-inflammatory cytokine IL-1β, GSDMD also mediates the secretion of fms-like tyrosine kinase 1 from macrophage/monocyte cell line THP-1, potentially contributory to preeclampsia pathology (43).

In a toll-like receptor (TLR)-dependent manner, GSDMD actively mediates the release of IL-1β from monocytes (44). Interestingly, monocytes can secrete extracellular vesicles to deliver active GSDMD to impact mesenchymal cell fate (45). As aforementioned, GSDMD is involved in the formation of NETs by neutrophils. Mechanistically, GSDMD can trigger mitochondrial pore formation in neutrophils, causing the leakage of mitochondrial DNA, which activates cGAS-STING signaling to stimulate NET formation (46). Additional to plasma membrane and mitochondria, GSDMD can migrate to azurophilic granules and autophagosomes of neutrophils to mediate IL-1β release (35). In aging neutrophils, cytoplasmic granules release neutrophil elastase, a neutrophil-specific serine protease, to cleave GSDMD at a distinct site for the induction of pore formation and death in neutrophils, resulting in anti-inflammatory effects (47). Meaningly, GSDMD can elicit pleiotropic effects (both pro- and anti-inflammatory effects), depending on the cleavage sites targeted by different proteases. Besides, GSDMD-mediated pyroptosis in macrophages facilities the secretion of microvesicles, encapsulating GSDMD-N-expressing mitochondria and mitochondrial ROS. These microvesicles in turn accelerate NET formation (48).

### 3.2 Cardiovascular cells

GSDMD, in a NLRP3/caspase-1-dependent manner, drives pyroptosis in endothelial cells. Histone deacetylase 11 promotes the NLRP3/caspase-1/GSDMD cascade by regulating E−26 transformation−specific−related gene (ERG) acetylation in human endothelial cells (49). Caspase-4/11-mediated GSDMD cleavage has been reported to contribute to pyroptosis, endothelial dysfunction, and subsequent pulmonary arterial hypertension (50). The longevity gene SIRT1 was found to be upstream to GSDMD-mediated pyroptosis in endothelial cells, where metformin treatment protects against LPS-induced endothelial pyroptosis and lung injury through SIRT1 upregulation (51). GSDMD was also shown to form mitochondrial pores in endothelial cells, causing the leakage of mitochondrial DNA, which activates cGAS Signaling and inhibits YAP-mediated endothelial regeneration (52). GSDMD has been implicated to modulate endothelial cell hyperactivation, a cell state facilitating the release of inflammatory factors. During vascular aging, protein kinase R activation promotes GSDMD cleavage, and hence endothelial pore formation and release of IL-1β and HMGB1, where these factors enhance phenotypic transformation of vascular smooth muscle cells (VSMCs) (53).

GSDMD-mediated pyroptosis in cardiomyocytes has been implicated in promoting myocardial ischemia/reperfusion (I/R) injury (54). GSDMD can also cause pore formation in cardiomyocyte mitochondria to mediate mitochondrial injury and mitophagy (55). During angiotensin II or pressure-overload-induced cardiac hypertrophy, GSDMD generates a positive feed-forward amplification loop through the mitochondria-STING axis. Briefly, GSDMD-N facilitates mitochondrial pore formation in cardiomyocytes, where the released mitochondrial DNA activates the STING/NLRP3/caspase-1 cascade to generate more GSDMD-N (56). Furthermore, GSDMD was found to form pores on ER membrane to activate ER stress by regulating the activity of FAM134B, an endoplasmic reticulum autophagy receptor, enhancing autophagy and apoptosis in doxorubicin-treated cardiomyocytes (57). The location of pore formation sites might be crucial for explaining the pleiotropic effects of GSDMD in different cell types.

### 3.3 Epithelial cells

MLKL-mediated necroptosis and GSDMD-mediated pyroptosis were observed in intestinal epithelial cells, where Fas-associated death domain (FADD), an adapter essential for caspase-8 activation, can inhibit both MLKL-mediated necroptosis and GSDMD-mediated pyroptosis to suppress intestinal inflammation (58). Besides, GSDMD plays a non-pyroptotic role by facilitating the secretion of IL-1β-encapsulating small extracellular vesicles from intestinal epithelial cells to mediate intestinal inflammation (59). Notably, GSDMD plays another non-pyroptotic role in intestinal epithelial cells by modulating mucus secretion in shaping intestinal homeostasis (60). Moreover, GSDMD modulates IL-33 secretion from mouse lung epithelial cells, where IL-33 plays a pivotal role in triggering airway inflammatory diseases (61). GSDMD is actively involved in mediating inflammation and epithelial-mesenchymal transition in pulmonary tissues (62). Recently, another less recognized N-terminal fragment of GSDMD (13 kD) has been identified, where such fragment results from caspase-3/7-mediated cleavage in intestinal epithelial cells upon exposure to dietary antigens. This 13-kD GSDMD fragment translocates into nucleus of intestinal epithelial cells to induce transcription of MHCII and CIITA for regulating immunity versus tolerance in small intestine (63). The presence of other less recognized GSDMD fragments and their potential biological functions require future extensive examination.

### 3.4 Other cells

The NLRP3/GSDMD axis was shown to modulate pyroptosis and inflammation in pancreatic β cells during hyperglycemia, implying the role of GSDMD in promoting the progression of diabetes mellitus (64). Pharmacological inhibition on NLRP3/caspase 1/GSDMD cascade by empagliflozin was shown to be beneficial against pancreatic damage during diabetes (65). GSDMD-mediated pyroptosis plays pivotal roles in hepatic homeostasis. GSDMD-mediated pyroptosis negatively modulates hepatocyte growth that GSDMD knockout significantly enhanced liver regeneration after 70% partial hepatectomy in mice (66). Direct GSDMD inhibition by phenethyl isothiocyanate, a natural compound present in cruciferous vegetables, can attenuate concanavalin A-induced hepatocyte pyroptosis and acute liver injury in mice (67). Pharmacological scavenging of mitochondrial ROS by quercetin, a dietary phytochemical presented in various vegetables, suppresses GSDMD-mediated pyroptosis in cultured human hepatocytes (68). Cleaved GSDMD was implied to accumulate on ER, leading to calcium leakage to the cytoplasm and resultant secretion of HMGB1-containing extracellular vesicles from hepatocytes (69). Meanwhile, GSDMD-mediated pyroptosis has been implicated in numerous types of cancer but with conflicting results (both pro- and anti-carcinogenic) depending on tumor cells used (70). Future extensive studies are required to elucidate the comprehensive role of GSDMD in different cancers at different stages for the development of potent anti-cancer therapies.

## 4 Role of GSDMD in inflammatory diseases

**Figure 5:**
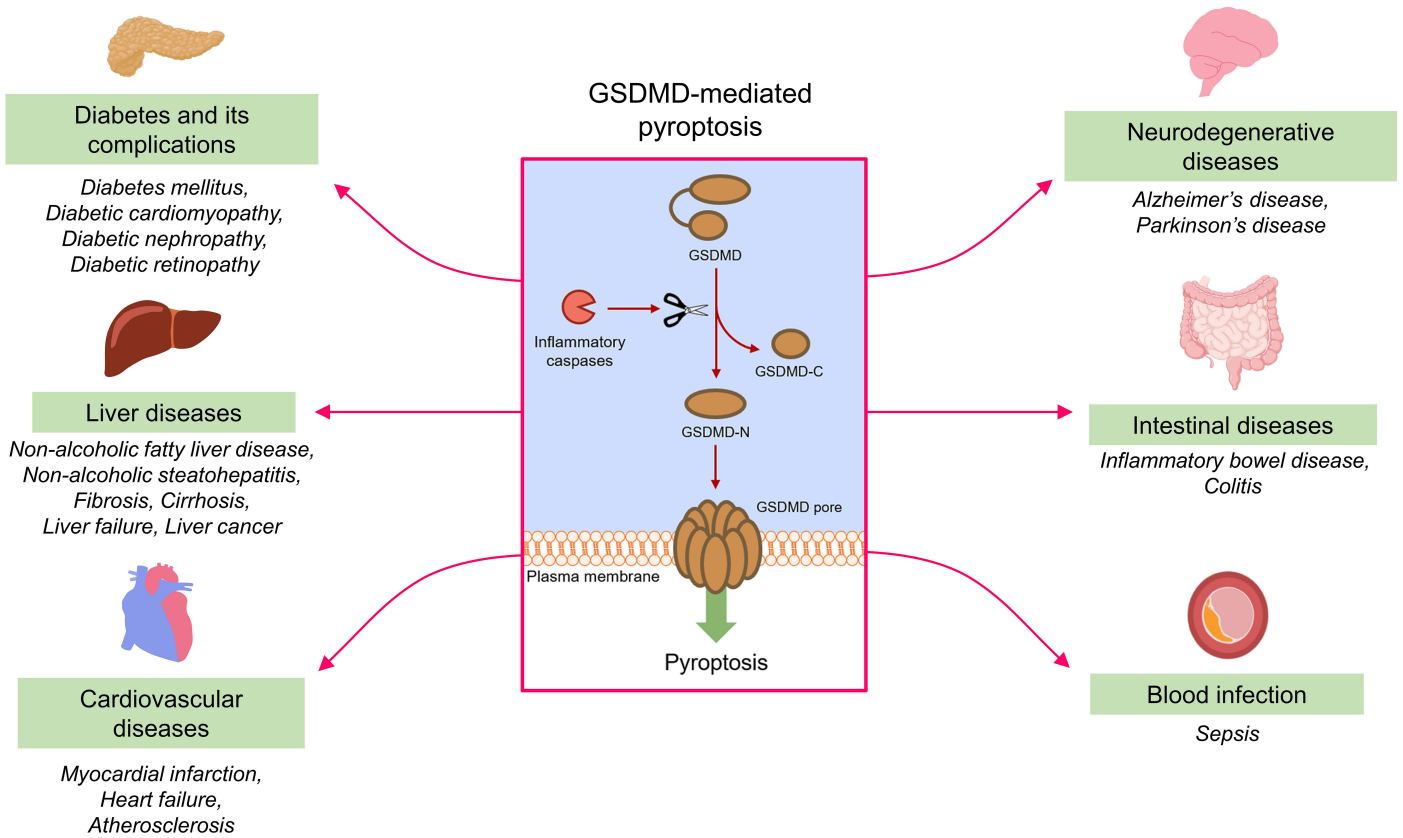
GSDMD-mediated pyroptosis and inflammatory diseases. Under the action of inflammatory caspases, GSDMD is cleaved into fragments containing NTD and CTD (GSDMD-N and GSDMD-C). GSDMD-N assembles to form permeability pores in plasma membrane to promote pyroptosis. GSDMD-mediated pyroptosis contributes to the onset and progression of different inflammatory diseases.

GSDMD plays a vital role in the onset and progression of various inflammatory diseases, including but not limited to diabetes mellitus, liver diseases, cardiovascular diseases, neurodegenerative disorders, intestinal diseases and blood infection. Pathophysiologically, GSDMD mediates pyroptosis to aggravate the progression of different inflammatory diseases (**Figure 5**).

### 4.1 Diabetes mellitus and its complications

GSDMD is involved in the pyroptosis-related inflammasome pathway to cause damage in pancreatic tissues during diabetes mellitus (65), where injured and dysfunctional pancreatic β cells are responsible for insulin deficiency and hence diabetes progression. Growing evidence has highlighted the roles of inflammation-associated pyroptosis and GSDMD in the pathogenesis of diabetes complications (71). Previous research reported that expression of pyroptosis-related proteins, such as GSDMD-N, NLRP3 inflammasome, caspase-1 and IL-1β, were upregulated in the cardiac tissues of diabetic mice (72). Moreover, higher levels of pyroptotic indicators, including GSDMD-N, caspase-1, caspase-11 and IL-1β were noted in doxorubicin-treated H9c2 cardiomyoblasts (73). Wang et al. has also suggested that the cleavage of GSDMD by caspase-1 is downstream to hyperglycemia-induced oxidative stress in H9c2 cardiomyoblasts (74). GSDMD-mediated pyroptosis was found downstream to mitochondrial dysfunction and ROS in diabetic cardiomyopathy mice in a cGAS/STING-dependent manner (75), however, we cannot exclude the possibility that GSDMD might generate a feedforward loop by forming pores on mitochondria. These findings implied the potential role of GSDMD in diabetic cardiomyopathy.

Diabetic nephropathy is one of the detrimental complications of diabetes mellitus and is considered as a sterile inflammatory disorder. The expression of pyroptosis-associated proteins, particularly GSDMD, caspase-1 and NLRP3, were found remarkably elevated in the renal tissues of diabetic mice (76). Notably, podocyte loss contributes to the progression of diabetic nephropathy (77). The expression of pyroptosis-associated proteins, such as GSDMD-N and NLRP3, were also found significantly augmented in podocytes upon hyperglycemic insult (78). Importantly, knockout of GSDMD attenuated podocyte loss in HFD/STZ-induced diabetic mice (79), hinting a pathophysiological role of GSDMD in the progression of diabetic nephropathy. Diabetic retinopathy causes severe visual damage and accumulated blindness in patients with diabetes mellitus. In human retinal pericytes, treatment with advanced glycation end product modified bovine serum albumin triggered the cleavage of caspase-1 and GSDMD, release of pro-inflammatory cytokines like IL-1β and IL-18, diminished cell viability and thereby progression of diabetic retinopathy (80). Moreover, hyperglycemia activated the NLRP3/caspase-1/GSDMD axis to cause pore formation, where GSDMD further aggravated pyroptosis in human retinal pericytes (81). These findings indicated the harmful role of GSDMD in retinal health.

### 4.2 Liver diseases

Non-alcoholic fatty liver disease (NAFLD) results from fat accumulation in liver, which predisposes the patients to non-alcoholic steatohepatitis (NASH), fibrosis, cirrhosis, liver failure and liver cancer. The pathophysiological process is accompanied by chronic inflammation in liver tissues. GSDMD plays a pivotal role in mediating pyroptosis during NAFLD progression. The caspase-11/GSDMD cascade was shown to promote hepatic macrophage pyroptosis and inflammation to aggravate NAFLD progression (82). Notably, GSDMD and GSDMD-N were upregulated in the liver of patients with NAFLD/NASH. Additionally, GSDMD-N level was positively correlated to fibrosis (83). These findings imply that GSDMD-N is a potential biomarker for NASH diagnosis. Mechanistically, GSDMD activity contributes to NASH progression by promoting pore formation, and the subsequent cytokine secretion from liver (e.g. IL-1β, TNF-α, and MCP-1), macrophage infiltration, and persistent activation of NF-κB signaling pathway (84). GSDMD-mediated pyroptosis is upstream to the inflammation and fibrosis in liver tissues during the development of NASH (85). In mice with acute liver failure, GSDMD knockout significantly alleviated inflammatory damage and improved the survival rate (86). Furthermore, GSDMD and GSDMD-N were found highly expressed in the liver from patients with hepatocellular carcinoma and metastatic hepatocellular carcinoma. RNA sequencing demonstrated that GSDMD knockout altered cyclic GMP-AMP synthase and immune-related pathways (87). These findings highlight the role of GSDMD in different stages of liver diseases.

### 4.3 Cardiovascular diseases

GSDMD participates in the development of multiple cardiovascular diseases by mediating pyroptosis in different cellular components of the cardiovascular system. In cardiomyocytes, cleavage of GSDMD causes the secretion of the pro-inflammatory cytokine IL-18 and augmented pyroptosis to promote myocardial I/R injury (54). Myocardial infarction refers to the decrease or shortage of blood flow to a heart portion, resulting in necrosis of heart muscles. During myocardial infarction, upregulated CXCR4 activates the NF-κB/GSDMD axis to cause damage to cardiomyocytes and impair heart function (88). Jiang et al. showed that GSDMD is required for the recruitment of neutrophils/monocytes to the infarcted mouse hearts, and GSDMD knockout dramatically attenuated myocardial injury after acute myocardial infarction (89). Heart failure refers to a chronic condition where the heart muscles fail to pump enough oxygen-rich blood to body parts. Myocardial remodeling is one of the major lesions in later stage of chronic heart failure. Pharmacological inhibition of GSDMD by the anti-inflammatory drug oridonin has been shown to lower the degree of cardiac remodeling both *in vitro* and *in vivo* (90). Cardiac hypertrophy often precedes the onset of heart failure. Pharmacological inhibition of GSDMD and GSDMD-mediated inflammation by DL-3-n-butylphthalide suppressed cardiac hypertrophy in the mouse model of transverse aortic constriction (91).

Atherosclerosis refers to the accumulation of lipid on artery walls and the chronic inflammation of arteries, underpinning ischemic heart disease and stroke (92). GSDMD knockout remarkably limited the development of atherosclerotic lesions in mice subjected to genetic inhibition of low density lipoprotein receptor (LDLR) (93). Recently, Puylaert et al. provided clues that GSDMD knockout did not alter the initiation of plaque formation in ApoE^-/-^ mice (94). Instead, GSDMD knockout could facilitate a switch from pyroptosis to apoptosis in macrophages, hence restraining the transition of atherosclerotic plaques to a vulnerable and inflammatory phenotype (94). Moreover, GSDMD was shown to disrupt cholesterol efflux from macrophages, and thereby promoting foam cell formation and atherogenesis (93). Dysfunction of endothelial cells initiates atherogenesis, where GSDMD-mediated pyroptosis promotes endothelial dysfunction (95). In endothelial cells, TNF-α treatment upregulated the expression of histone deacetylase 11 (HDAC11), which subsequently ignited the NLRP3/caspase-1/GSDMD signaling cascade to mediate pyroptosis and inflammation (49). Cell deaths in the vasculature, particularly the VSMCs, promote atherosclerotic progression. In VSMCs, GSDMD has been found to mediate pyroptosis and inflammation in a melanoma 2-dependent manner (96). These findings indicate the contributory roles of GSDMD in different cellular components of cardiovascular system during atherosclerotic progression.

### 4.4 Neurodegenerative diseases

Substantial amount of evidence has identified the tight connection between pyroptosis and the development of neurodegenerative diseases, such as Alzheimer’s and Parkinson’s diseases (97). In the cerebrospinal fluids from patients with Alzheimer’s disease, the levels of GSDMD and T-Tau were significantly higher than those from healthy controls (98). One of the pathological features of Alzheimer’s disease is the formation of senile plaque due to the deposition of β‐amyloid in extracellular space. β‐amyloid has been demonstrated to induce pyroptosis in mouse cortical neurons, in which the expression levels of pyroptotic proteins like GSDMD, GSDMD-N, NLRP3 inflammasome and caspase-1 were upregulated (99). β‐amyloid-induced oxidative stress was found upstream to GSDMD-mediated pyroptosis in neuronal cells (100). Sevoflurane, the frequently used anesthetic, could activate the NLRP3/caspase-1/GSDMD cascade, and increase tau phosphorylation and β‐amyloid deposition, hence promoting the progression of Alzheimer’s disease (101). Like Alzheimer’s disease, GSDMD plays a pathological role in Parkinson’s disease. NLRP3/caspase-1/GSDMD pathway was shown to contribute to neuroinflammation in mouse model of Parkinson’s disease induced by N-methyl-4-phenyl-1,2,3,6-tetrahydropyridine (MPTP) (102). In a TLR4-dependent manner, MPTP activated NLRP3/caspase-1/GSDMD cascade in mouse model of Parkinson’s disease (103). In patients with Parkinson’s disease, GSDMD and GSDMD-N were found present in the extracellular vesicles isolated from plasma (104). These recent findings suggest the role of GSDMD in neurodegenerative diseases.

### 4.5 Other diseases

In patients with IBD and experimental colitis, GSDMD is highly expressed in intestinal epithelial cells. Notably, GSDMD was shown to guide the release of IL-1β-containing extracellular vesicles from intestinal epithelial cells, hinting the pathological role of GSDMD in intestinal inflammation (59). Dysregulation in microbiome, particularly commensal Escherichia coli, caused GSDMD activation to exacerbate colitis development by boosting IL-18 release from colon (105). GSDMD-mediated macrophage pyroptosis aggravates experimental colitis in mice (106). However, Ma et al. provided clues to suggest that GSDMD acts as a negative regulator of cyclic GMP–AMP synthase-dependent inflammation in macrophages, therefore conferring protective effect against colitis (107). Further study shall clarify the differential roles of GSDMD in different cell types. Sepsis refers to the severe organ dysfunction triggered by a dysregulated host response to infection, and a multifaceted disruption of the immunological balance between inflammation and anti-inflammation (108). GSDMD activity was observed in the neutrophils isolated from septic humans and mice. During sepsis, caspase-11/GSDMD pathway was activated to control the release of neutrophil extracellular traps by neutrophils (109), implying GSDMD as a therapeutic target against sepsis.

## 5 Clinical studies on GSDMD

Various clinical studies have correlated GSDMD level to severity and progression of different diseases. Clinically, fluctuation in GSDMD levels might potentially be diagnostic and prognostic factors in inflammatory diseases and events (**Table 1**).

**Table 1:** GSDMD studies in patients.

Diseases/Events	Tissue	GSDMD level	Ref.
Myocardial I/R injury	Serum	↑	(54)
Adult-onset Still’s disease	Serum	↑	(111)
Anti-N-methyl-D-aspartate receptor encephalitis	Serum	↑ (before treatment) ↓ (after treatment)	(113)
Parkinson’s disease	Plasma	↑	(104)
NAFLD/NASH	Liver	↑	(83)
Alzheimer’s disease	Cerebrospinal fluid	↑	(98)
Inflammatory bowel disease, experimental colitis	Intestinal epithelium	↑	(59)
Spontaneous labor at term	Amniotic fluid, chorioamniotic membrane	↑	(114)

↑ means "increased" ↓ means "decreased"

In patients exhibiting myocardial I/R injury, elevated serum levels of GSDMD were noted (54), implying GSDMD to be a potential biomarker and therapeutic target for the evaluation and treatment of myocardial I/R injury. Adult-onset Still’s disease (AOSD) refers to the rare systemic autoinflammatory disease, where the patients experience fevers, joint pain, and salmon-colored bumpy rash (110). In patients with AOSD, increased serum levels of GSDMD-N and IL-18 were detected, highlighting inflammasome activation and GSDMD-mediated pyroptosis in monocytes and macrophages (111). Anti-N-methyl-D-aspartate receptor (NMDAR) encephalitis refers to the rare autoimmune disorder in central nervous system, characterized by epilepsy, movement disorders, psychobehavioral changes and cognitive decline (112). Importantly, serum levels of GSDMD were remarkably higher in patients with anti-NMDAR encephalitis than those in healthy controls (113). A further 3-month follow-up evaluation revealed that serum GSDMD levels in patients with anti-NMDAR encephalitis decreased significantly post-treatment (113), implying that GSDMD might be a potential prognostic marker. In patients with Parkinson’s disease, GSDMD was also present in the extracellular vesicles isolated from plasma (104).

GSDMD levels in other tissues and body fluids might also serve as diagnostic and prognostic indicators. In patients with NAFLD/NASH, the levels of GSDMD and GSDMD-N were elevated in the liver tissues. Additionally, hepatic GSDMD-N levels were positively correlated to NAFLD activity score and fibrosis (83). Histological examination of GSDMD level in liver tissues might facilitate the severity assessment of liver diseases. Notably, in the cerebrospinal fluids from patients with Alzheimer’s disease, the levels of GSDMD was remarkably higher than those from healthy controls, implying that GSDMD might be a diagnostic biomarker for Alzheimer’s disease (98). As mentioned, GSDMD is upregulated in intestinal epithelial cells in patients with IBD and experimental colitis (59). In addition, GSDMD can be the *in vivo* clue indicating pyroptosis in spontaneous labor at term. In women who underwent spontaneous labor at term, GSDMD levels in the amniotic fluid and chorioamniotic membranes were higher than those without labor (114). Such finding might suggest that inflammasome-induced pyroptosis is involved in the physiological process of labor at term. Further study might uncover whether fluctuated GSDMD levels in amniotic fluid and chorioamniotic membranes are correlated to preterm labor and birth.

## 6 Therapeutic potential of GSDMD inhibitors

GSDMD inhibition shall be an attractive therapeutic strategy to counteract inflammation and hence inflammatory diseases. In recent years, a number of GSDMD inhibitors have been identified to restrain GSDMD-mediated pyroptosis through different mechanisms (**Table 2**).

**Table 2:** GSDMD inhibitors and their mechanism.

GSDMD inhibitors	Nature	Mechanism	Ref.
Necrosulfonamide	Necroptosis inhibitor	• Inhibit the oligomerization of GSDMD-N by binding to C191 amino acid	(116)
Bay 11-7082	NF-κB inhibitor	• Inhibit GSDMD oligomerization by binding to C191/192 residue	(117)
Disulfiram	Drug against alcohol addiction	• Inhibit GSDMD oligomerization by binding to C191/192 residue • Modify C191/192 residue of GSDMD covalently	(117, 118)
LDC7559	NETosis inhibitor	• Inhibit GSDMD activity	(32)
Dimethyl fumarate	Nrf2 activator	• Cause GSDMD succination • Hinder the interaction between GSDMD and caspases • Restrain the oligomerization and pyroptosis-inducing capacity of GSDMD	(120)
GI-Y1	Novel GSDMD inhibitor	• Target the Arg7 residue of GSDMD-N • Inhibit GSDMD-mediated lipid binding and pore formation • Suppress the binding of GSDMD-N to mitochondria	(121)
Punicalagin	Phenolic compound	• Prevent the insertion of GSDMD-N into plasma membrane • Limit IL-1β release	(122)
Ac-FLTD-CMK	Caspase inhibitor	• Inhibit GSDMD cleavage • Limit IL-1β release	(123)

Necrosulfonamide (NSA) is an inhibitor of necroptosis. NSA specifically blocks mixed-lineage kinase domain-like protein, which is the critical signaling molecule of necroptosis (115). Until recently, NSA has also been considered as a GSDMD inhibitor and NSA can potentially inhibit the oligomerization of GSDMD-N by docking to C191 amino acid (116). Bay 11-7082, a NF-κB inhibitor, and disulfiram, a drug commonly used against alcohol addiction, were found to be potential GSDMD inhibitors (16, 117). Similar to NSA, Bay 11-7082 and disulfiram also function as inhibitors of GSDMD oligomerization by binding to C191/192 residue (117, 118). More importantly, disulfiram covalently modifies C191/192 residue of GSDMD in both human and mice to block pore formation, hence limiting IL-1β release (117). However, Bay 11-7082 and disulfiram might target other pyroptosis-related proteins, implying the lack of specificity (119). Further efforts are needed to optimize the specificity of these two drugs before *in vivo* application.

LDC7559 is an inhibitor of neutrophil extracellular traps. In a large screening of library covering over 182,000 compounds, LDC7559 was identified to bind to GSDMD hindering NETosis (32). Mechanistically, LDC7559 can abrogate the toxicity of GSDMD-N in both human and mice, demonstrating a direct inhibitory effect of LDC7559 on GSDMD activity (32). Dimethyl fumarate (DMF), a Nrf2 activator used to treat multiple sclerosis, is also a potent GSDMD inhibitor. Humphries et al. provided evidence that DMF causes GSDMD succination by reacting with GASDMD at critical cysteine residues. Importantly, GSDMD succination hinders the interaction between GSDMD and caspases, restraining the oligomerization and pyroptosis-inducing capacity of GSDMD (120). In 2023, a new GSDMD inhibitor GI-Y1 identified by virtual and pharmacological screening was shown to inhibit GSDMD-mediated lipid-binding, pore formation and mitochondrial binding by targeting Arg7 residue of GSDMD-N in cardiomyocytes, implying the potentially cardioprotective effect of GI-Y1 against myocardial I/R injury (121).

Punicalagin, a phenolic compound reported to elicit antioxidant and anti-inflammatory effects, is another potent GSDMD inhibitor that prevents the insertion of GSDMD-N into plasma membrane of macrophages through its antioxidant effect on reactive thiols. Coherently, IL-1β release from macrophages were also decreased (122). However, whether punicalagin inhibits the cleavage of GSDMD, or elicits its effect independently of GSDMD, requires further study. Ac-FLTD-CMK, a selective inhibitor of caspases, has been suggested to be another potent GSDMD inhibitor. In macrophages, treatment with Ac-FLTD-CMK inhibited GSDMD cleavage, IL-1β release and pyroptosis (123). Nevertheless, whether these potent GSDMD inhibitors target pyroptosis in other non-immune cells to retard disease progression requires future extensive study. Further investigations with the aid of virtual and pharmacological screening tools shall facilitate the identification of more potent GSDMD inhibitors.

## 7 Other therapeutic agents against GSDMD-mediated pyroptosis

Certain studies have evaluated the potential of some therapeutic agents against GSDMD-mediated pyroptosis in different disease models. During progression of alcoholic steatohepatitis, GSDMD pore formation is critical to the excessive release of IL-1β from ethanol or acetaldehyde-stimulated macrophages (124). Luan et al. developed a hepatocyte-specific nanobiologics for prolonged expression of Interleukin-1 receptor antagonist and IL-1β blockade in liver (124). In other words, such nanobiologics counteracted GSDMD-mediated release of pro-inflammatory cytokine IL-1β to retard progression of alcoholic steatohepatitis. In addition, in mice with acute liver injury, 3-day administration of phenethyl isothiocyanate (PEITC), a natural compound found in cruciferous vegetables, alleviated hepatic pyroptosis and liver injury (67). Meanwhile, ellagic acid, a polyphenolic compound present in many fruits, was shown to protect mice against hepatic ischemia–reperfusion injury by targeting caspase-1/GSDMD pathway (125).

GSDMD can accumulate in ER to cause ER stress in pancreatic acinar cells. A recent study showed that diosgenin derivative D (Drug D) attenuated pancreatic inflammation by limiting GSDMD accumulation in ER of acinar cells (126). Furthermore, *in vivo* and *in vitro* studies showed that the anti-inflammatory drug oridonin can attenuate cardiac remodeling by suppressing GSDMD-mediated pyroptosis and inflammation in cardiomyocytes (90). In septic mice, administration of fudosteine, a cysteine derivative, could suppress pyroptosis by targeting the TXNIP/NLRP3/GSDMD signaling cascade (127). Helicobacter pylori (*H. pylori*) infection is closely related to the development of neuroinflammation and the progression of depression. Rapamycin treatment significantly lowered GSDMD expression in hippocampus and inhibited depression-like behavior in *H. pylori*-infected mice (128). However, further extensive clinical studies are urged to evaluate the safety and efficacy of these therapeutic agents in human patients. of virtual and pharmacological screening tools shall facilitate the identification of more potent GSDMD inhibitors.

## 8 Conclusion and perspectives

GSDMD plays a critical role in the initiation and progression of various inflammatory diseases. GSDMD mediates pyroptosis and inflammation in multiple cell types during disease progression. Certain clinical studies have suggested GSDMD to be potential diagnostic and prognostic marker in different diseases. Certain preclinical studies have identified potential GSDMD inhibitors and other therapeutic agents to counteract pyroptosis and inflammation mediated by GSDMD.

However, a number of questions remain to be addressed in future investigation. Of note, certain GSDMD inhibitors, like NSA, Bay 11-7082 and disulfiram, target the C191/192 residue to prevent GSDMD oligomerization and pore formation, thereby limiting pyroptosis and inflammation. The exact role of the C191/192 residue of GSDMD requires further investigation. In addition, many preclinical and clinical studies are focusing on the pathophysiological role of GSDMD in immune cells, such as monocytes and macrophages. Future studies shall investigate the role of GSDMD in other non-immune cells in the context of different diseases. Serum GSDMD levels increase during myocardial I/R injury. It would be interesting to clinically correlate the elevation in serum GSDMD level with the progression of other atherosclerotic cardiovascular diseases, since GSDMD has been shown to contribute to atherosclerotic plaque formation.

**Figure 6:**
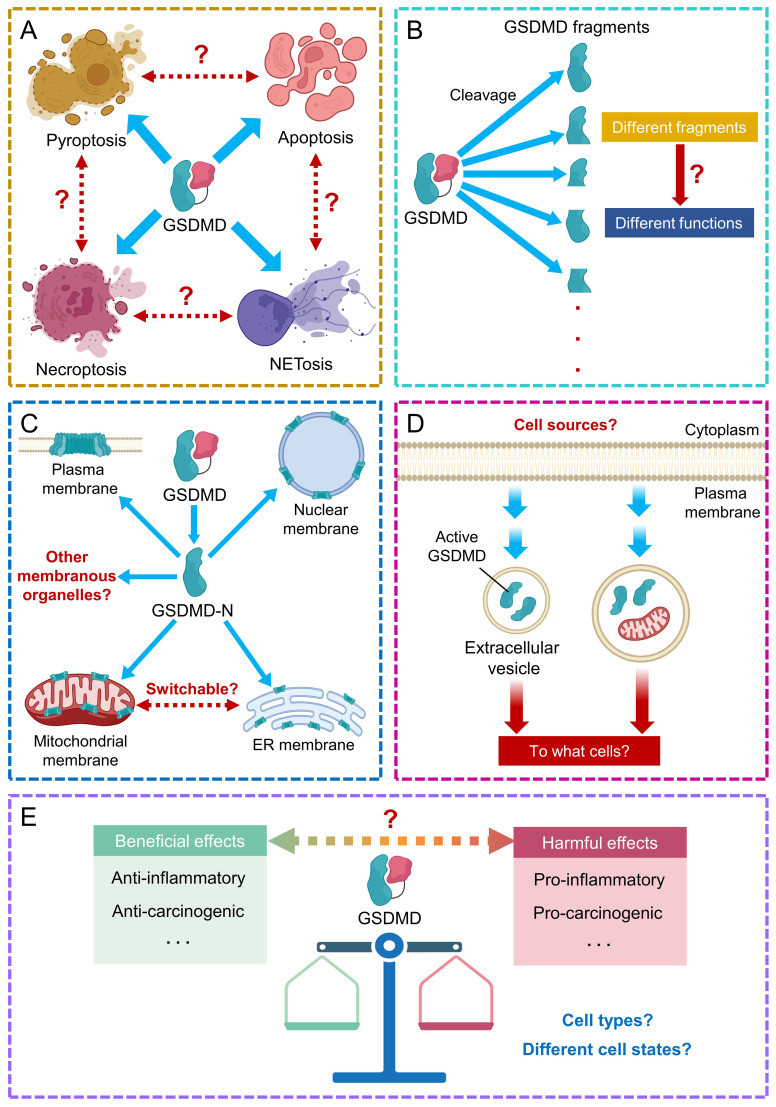
Future perspectives on GSDMD research. **(A)** The role of GSDMD in the crosstalk between different types of cell death requires further study. **(B)** Identification of other GSDMD fragments and their associated functions requires further efforts. **(C)** The comprehensive consequences downstream to GSDMD-mediated pore formation on recognized and other membranous organelles/structures need further investigation. **(D)** Potential cell sources and target cells of GSDMD-encapsulating extracellular vesicles remain largely unexplored. **(E)** The pleiotropic effects of GSDMD in different cell types at different cell states remain elusive.

Furthermore, the comprehensive role of GSDMD in the crosstalk between different types of cell death requires further extensive investigation. Different proteases cleave GSDMD at distinct sites, so future identification of the collection of GSDMD fragments and their biological functions shall benefit our understanding towards the mechanism of inflammatory diseases, and the development of more anti-inflammatory therapies. The sites of pore formation by GSDMD are also important information for understanding the mechanism of inflammatory diseases. Strategies that can reduce pore formation on membranous structures, including plasma and nuclear membranes, ER and mitochondria, or can shift the sites of perforation by GSDMD may provide new therapeutic opportunities. Identification of additional tissues and cells that could release GSDMD-containing extracellular vesicles shall broaden our understanding on the role of GSDMD in cell-cell communication during disease progression. GSDMD-mediated pyroptosis elicits pleiotropic roles in normal cells (both pro- and anti-inflammatory) and tumor cells (both pro- and anti-carcinogenic). Efforts are needed to develop specific GSDMD modulators to exert beneficial and protective effects on desired cells (**Figure 6**).

Collectively, further efforts are still required to enhance our understanding towards the detailed mechanistic network of GSDMD-mediated pyroptosis and inflammation, and to narrow the gap between preclinical and clinical studies.
